# Mechanism and Therapeutic Prospect of miRNAs in Neurodegenerative Diseases

**DOI:** 10.1155/2023/8537296

**Published:** 2023-11-23

**Authors:** Ya-Min Ma, Lan Zhao

**Affiliations:** ^1^Acupuncture and Massage Department of Nanyang Traditional Chinese Medicine Hospital, Wo Long District, Nanyang City 473000, China; ^2^Tianjin Key Laboratory of Acupuncture and Moxibustion, First Teaching Hospital of Tianjin University of Traditional Chinese Medicine, Xiqing District, Tianjin 300381, China

## Abstract

MicroRNAs (miRNAs) are the smallest class of noncoding RNAs, which widely exist in animals and plants. They can inhibit translation or overexpression by combining with mRNA and participate in posttranscriptional regulation of genes, resulting in reduced expression of target proteins, affecting the development, growth, aging, metabolism, and other physiological and pathological processes of animals and plants. It is a powerful negative regulator of gene expression. It mediates the information exchange between different cellular pathways in cellular homeostasis and stress response and regulates the differentiation, plasticity, and neurotransmission of neurons. In neurodegenerative diseases, in addition to the complex interactions between genetic susceptibility and environmental factors, miRNAs can serve as a promising diagnostic tool for diseases. They can also increase or reduce neuronal damage by regulating the body's signaling pathways, immune system, stem cells, gut microbiota, etc. They can not only affect the occurrence of diseases and exacerbate disease progression but also promote neuronal repair and reduce apoptosis, to prevent and slow down the development of diseases. This article reviews the research progress of miRNAs on the mechanism and treatment of neurodegenerative diseases in the nervous system. This trial is registered with NCT01819545, NCT02129452, NCT04120493, NCT04840823, NCT02253732, NCT02045056, NCT03388242, NCT01992029, NCT04961450, NCT03088839, NCT04137926, NCT02283073, NCT04509271, NCT02859428, and NCT05243017.

## 1. Introduction

Neurodegenerative diseases mainly occur in middle-aged and elderly people, and their incidence rate and mortality are increasing day by day, which has become a major health problem worldwide [1]. Its main feature is irreversible degeneration of neurons and (or) myelin sheath in the central or peripheral nervous system. The etiology is highly complex, which is closely related to age. The global elderly population is increasing, and the prevalence of neurodegenerative diseases is also increasing [2]. In clinical practice, Alzheimer's disease (AD), Parkinson's disease (PD), Huntington's disease (HD), and amyotrophic lateral sclerosis (ALS) are common. Although many researchers have made great contributions to the pathological mechanism and treatment strategies of neurodegenerative diseases, clear diagnosis and effective treatment plans need to be further explored [3]. At present, the discovery of miRNAs plays an important role in the diagnosis, treatment, and physiological and pathological regulation of neurological diseases in the nervous system [4].

**Figure 1:**
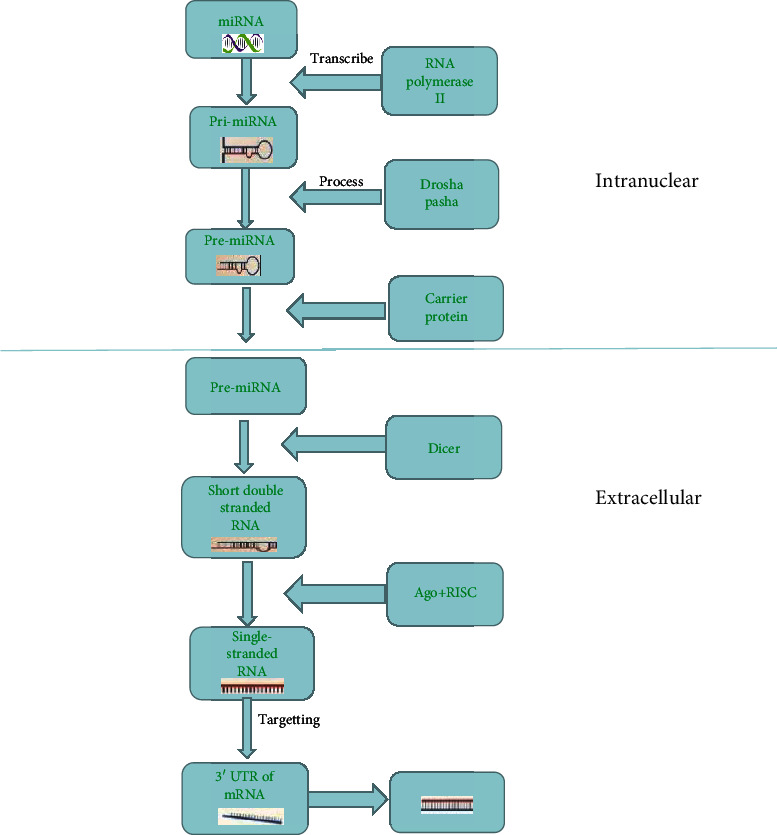
The production of mature miRNAs and their inhibition of mRNA transcription and translation processes.

miRNAs are small noncoding single-stranded RNA molecules; it is approximately 20-24 nucleotides long, which specifically bind to the 3′-untranslated region (3′-UTR) of the target mRNA and are important posttranscriptional regulators of gene expression, leading to their degradation or inhibition of protein translation [5]. The first confirmed miRNAs were lin-4 found in nematodes for the first time, and then, researchers found a large number of miRNAs in humans, fruit flies, plants, and other biological species [6]. About 2000 miRNAs have been found in the human genome. In the nucleus of animals, the primary transcript is transcribed by RNA polymerase II into pri-miRNA and then processed into pre-miRNA with hairpin structure consisting of 70 nucleotides under the action of nuclease Drosha and its cofactor Pasha. Finally, the pre-miRNA is transferred from the nucleus to the cytoplasm by a carrier protein, which is split into short double-stranded RNA of about 22 nucleotides by another nuclease Dicer, and then, this short double-stranded RNA is introduced into the RISC silencing complex containing Ago protein. One mature single-stranded RNA is retained in the RISC silencing complex and is combined to the site of its complementary mRNA to regulate gene expression through base pairing [7]. There is literature showing that each miRNA may regulate the expression of thousands of proteins, leading to their ability to affect the entire biological process [8]. The transcription and translation process of miRNAs inhibiting mRNA is shown in Figure 1.

In recent years, miRNAs have been found to express abundantly and variously in the mammalian central nervous system, participating in the development of neurons, synaptic plasticity, and neurotransmission, as well as in the degeneration of the Purkinje cells or midbrain dopaminergic neurons, cortex, or hippocampus neurons [9–11]. In the study, it was found that the targets of miR-101 are all key proteins that regulate synaptic growth and are related to the transcription of RNA polymerase II promoter processes and chemical synaptic transmission processes. They are considered important regulatory factors that inhibit the balance between neurons and excitatory neurons [12, 13]. It is known that neural networks can regulate the excitation and inhibition of neurons, prevent and treat pathological changes such as neural hyperexcitation and memory loss, and balance excitation to ensure the normal operation of neural circuits. Lippi et al. found that miR-101 plays an important role in this role. Through in vivo model research, Lippi et al. also believe that during the window period of neural development, miR-101 can target hundreds of different mRNAs and regulates multiple postnatal developmental programs. Repression of one target, NKCC1, activates g-aminobutyric acid signaling and limits dendritic growth. Kif1a and Ank2 are targeted to prevent excessive synapse formation. They jointly regulate the neural network, balance neuronal excitation, and prevent the occurrence of neurological diseases [14, 15]. Through detection, it was found that miRNAs are differentially expressed in AD brain tissue. For example, compared with the normal control group, miR-129-5p, miR-132-5p, and miR-138-5p are significantly downregulated; however, miR-195-5p and hsa-miR-101-3p are significantly upregulated in the entorhinal cortex (EC), indicating that these miRNAs may be potential therapeutic targets for AD [16, 17]. In the study of Drosophila as a genetic model, moderate knocking down of Rac1 in neurons can lead to neurodegeneration in Drosophila. However, overexpression of hsa-miR-101-3p in the EC of AD brain leads to downregulation of the mRNA expression level of RAC1 gene, leading to AD pathology [17]. The literature suggests that miR-101 is low expressed in the spinal tract of mouse brain neurons, and it is downregulated in the hippocampus of AD mice, which may be hyperphosphorylated through AMPK, that the continuous increase in *β*-amyloid (A*β*) 42 levels reduces the spatial orientation and target recognition abilities of mice [18, 19]. Another study found that miR-101 has the highest expression level in NT2 neurons, and binding to specific sites of the prerequisite protein 3′-UTR can reduce the expression of APP in human cells [20]. Previous studies have shown that miR-19, miR-101, and miR-130 directly bind to ataxia protein 1 (ATXN1) 3′-UTR, inhibiting the amplification of translated CAG repeat sequences in TXN1, thereby inhibiting the translation of ATXN1 protein, reducing the accumulation of TXN1 error proteins in the Purkinje cells, reducing cell death, and possibly improving neurodegeneration [21]. Similarly, in HD, Ras homolog enriched in striatum (Rhes) affects the development of HD pathology through various signaling pathways in the body. miR-101 can bind to the 3′-UTR of Rhes mRNA, inhibit the expression of Rhes, and prevent the progression of HD disease [22, 23]. Autophagy vesicles are not common in the normal brain but are enriched in the AD brain. It is known that miRNAs play a key role in autophagy regulation. In vitro and in vivo, miRNA-101a has been explored to be low expressed in AD patients and APPswe/PS1DE9 mice, possibly regulating cell autophagy and improving neuronal function through its target MAPK pathway [24]. In neurodegenerative diseases, overexpression of a single *α*-synaptic nucleoprotein (*α*-syn) protein in the brain does not produce toxicity or abnormal aggregation. However, overexpression in combination with miR-101-3p can cause neurotoxicity and abnormal protein aggregation, leading to pathological changes in PD. It has been further confirmed that under physiological conditions, the target gene of miR-101-3p, S-phase kinase-associated protein 1 (SKP1), directly interacts with PLK2, promoting ubiquitination of PLK2 and promoting autophagic degradation of A. Overexpression of PLK2 can partially alleviate MPP+-induced neurotoxicity. Overexpression of miR-101-3p in PD neurons may inhibit the expression of SKP1. It has been further confirmed that under physiological conditions, the target gene of miR-101-3p, S-phase kinase-associated protein 1 (SKP1), directly interacts with PLK2, promoting ubiquitination of PLK2 and promoting autophagic degradation of *α*-Syn. Overexpression of PLK2 can partially alleviate MPP+-induced neurotoxicity. Overexpression of miR-101-3p in PD neurons may inhibit the expression of SKP1. Overexpression of miR-101-3p in PD neurons may inhibit the ubiquitination of PLK2 by inhibiting the expression of SKP1, leading to abnormal accumulation and aggregation of *α*-Syn, exacerbating PD pathology [25]. miR-101-3p can also be used as an lncRNA-T199678 target in PD to reduce *α*-syn-induced oxidative stress levels and prevent cell cycle redistribution and neuronal apoptosis [26, 27]. Additionally, miR-101 is overexpressed and reduced the accumulation of *α*-syn in oligodendrocytes, restore autophagy deficiency function, and improve demyelination and neurodegeneration [28]. According to the literature, the levels of miR-339-5p, miR-590-5p, miR-34a, miR-326, miR-155, miR-191-5p, etc., in the AD mouse model are all reduced. They may inhibit cell cycle reentry by inhibiting the target protein. According to literature, levels of miR-339-5p, miR-590-5p, miR-34a, miR-326, and miR-191-5p were all reduced in the AD mouse model. Studies have shown that their overexpression can improve the progression of AD and protect neurons. Among them, miR-339-5p improves mushroom spine loss and calcium dyshomeostasis by inhibiting target protein neuropeptides (Nnat); miR-590-5p inhibits pelino-1 (PELI1) and reduces phosphorylation of p38 MAPK and ERK1/2; miR-34a targeted the 3′-UTR region of the cyclin D1 gene to inhibit cell cycle reentry; miR-326 inactivates the JNK signaling pathway through Vav guanine nucleotide exchange factor 1 (VAV1), reducing accumulation of A *β*; and miR-191-5p reduces the activity of ERK1/2 and p38 MAPK by targeting upstream MAP3K12 effectors, to reduce the apoptosis rate of neuronal cells [29, 30, 31]. HMDD database showed that miRNA-gene network identified 11 miRNAs targeting HTT genes, including miR-34b-3p, miR-128-5p, miR-196a-5p, miR-34a-5p, and miR-338-3p, and interacting with HD-related genes. The interaction between HD-related genes and HTT is involved in cell apoptosis, energy metabolism, embryonic development, etc. Therefore, miRNAs may be used as biomarkers and potential therapeutic targets for diagnosing HD diseases. Inhibition of miR-128 can also improve the degradation of A*β* (1-42) and maintain the dynamic balance of A*β* to improve the clinical manifestations of AD [32, 33, 34]. A miRNA can have multiple target genes, and multiple miRNAs regulate the same target gene. This characteristic of miRNAs may determine their tendency to aggregate in the genome and participate in the occurrence and development of similar diseases. For example, miR-124-3p is abnormally expressed in AD, ALS, and PD, while hsa-miR-549a is upregulated in HD patients and can regulate five target genes. The interaction between miR-20a-5p, miR-17-5p, and miR19a-3p jointly regulates similar mechanisms of protein aggregation, mitochondrial dysfunction, and axonal transport defects [35, 36, 37]. Therefore, in degenerative disease such as AD, PD, ALS, and HD, miRNAs may affect neurodegeneration by activating microglia, promoting inflammation and protein aggregation, and inhibiting autophagy [38, 39]. At the same time, it was found that miRNAs are biomarkers for various diseases such as neurodegenerative diseases, which can serve as effective tools for diagnosing diseases [40]. GEO2R analysis shows low expression of miR-374a-5p and miR-335-5p in PD, which has diagnostic value [41].

This article introduces the mechanism of miRNAs in treating neurodegenerative diseases by regulating the expression of miRNAs to mobilize the human immune system, signal pathways, and stem cells and to regulate the repair of human brain injured neurons and improve the clinical symptoms of neurodegenerative diseases, as well as the limitations and challenges of miRNAs in clinical application [4, 42].

## 2. Effect of miRNAs on Neurodegenerative Diseases

### 2.1. miRNAs Regulate Immune System and Affect Neurodegenerative Diseases

According to statistics, the immune system is one of the main reasons affecting neurodegenerative diseases. It is well known that the cells that make up the brain include neurons, astrocytes, microglia, oligodendrocytes, and endothelial cells. When the brain is damaged, they produce inflammatory reaction to protect the brain from damage. At the same time, they are also inflammatory factors that cause brain and nerve damage, forming a vicious circle in the brain [43]. Microglia is one of the first batch of activated immune cells in the process of inflammatory reaction, belonging to megaphagocytic cells. It can clear debris, release neurotrophic factors, reduce neurotoxic substances, protect nerves, and maintain the stability of the central nervous system. However, abnormally activated microglia secrete a large amount of glutamate, damaging nerve cells [44]. Astrocytes and microglia are the main immune components of the central nervous system. Astrocytes gather and secrete IL-10 and TGF-*β* at inflammatory and injured areas; both of them are innate immunity and adaptive immunosuppressants, which inhibit inflammatory reaction and protect neurons from damage. Oligodendrocytes will produce immune response demyelinating in inflammatory environment, showing demyelinating lesions and triggering ALS [45–47] (Table 1).

According to research, astrocytes and activated microglia release exosomes and extracellular microbubbles, which contain rich miRNAs. These miRNAs are significantly upregulated in AD, ALS, HD, PD, and other neuroinflammatory diseases, promoting neuroinflammation, inducing complement activation, destroying innate immune signal transduction, and aggravating the disease [48]. It is reported that most neurodegenerative diseases are caused by heredity, environmental changes, aging, and other factors, usually caused by mutation of multiple genes [49, 50]. The abnormal accumulation of A*β* and *α*-syn is the main cause of degenerative disease such as AD and PD. These misfolding proteins activate neurons and M1-type microglia, produce inflammatory mediators, lead to neuronal damage and apoptosis, and also transform peripheral white blood cells into M2-type microglia to eliminate A*β* and *α*-syn and other neurotoxic substances to reduce neurotoxicity [51]. In addition, scholars have learned from weighted gene coexpression network analysis (WGCNA) that the immune and microglial modules are prominent in the gene coexpression network constructed in the brain tissue of late onset AD patients. At the same time, it has also revealed that the specificity of astrocytes and the enrichment of microglial modules are related to abnormal Tau protein aggregation. Therefore, the immune regulatory system is crucial to the occurrence and development of neurodegenerative diseases [52].

miRNAs exist in the entire immune system. It is known that miRNAs mediate gene expression regulation by binding with mRNA to inhibit the expression of proteins. The study shows that microglia can eliminate the abnormal accumulation of A*β* in the brain of AD mice through autophagy. Mirc1/mir-17-92 cluster reduces the autophagic ability of microglia by reducing the expression of autophagic receptor NBR1 protein; thus, weakening microglia's response to A*β* clearance promotes disease progression [53]. In addition to the abnormal accumulation of A*β* being the main cause of AD, neuroinflammation is another major cause of its onset. Glial cell activation occurs in the early stages of AD, and some speculate that it may occur in A*β* before stacking. The immune-related DEOSGs and hub genes were identified by weighted gene coexpression network analysis and protein-protein interaction analysis. Nine hub genes (CCK, CNR1, GAD1, GAP43, NEFL, NPT, PENK, SST, and TAC1) were associated with oxidative stress and immune response in the pathogenesis of AD. The expression of hsa-miR-27a-3p is downregulated in cerebrospinal fluid of AD patients, accompanied by high levels of tau protein and low levels of A*β*. It has been studied that hsa-miR-27a-3p may be most closely related to five hub genes, including CNR1, SST, PENK, CCK, and NEFL [54]. The miRNAs isolated from peripheral blood neutrophils in AD patients, such as miR-210, miR-20b-5p, and miR-126-5p, were significantly unbalanced compared with the healthy control group without systemic infection, suggesting that neutrophils may mediate kynurenine dysfunction and cognitive impairment caused by inflammation through the miRNAs they secrete [55]. A large number of previous studies have suggested that miRNAs have disordered gene expression in PD. Recently, the application of ingenuity pathway analysis found that the differential expression of miRNAs (miR-1275 and miR-432-5p) and a downregulated miR-99a-5p directly target the SMAD 2/3 signal pathway in PD. It has been determined that SMAD3 signals regulate the function of microglia cells, maintain the development and stability of microglia cells, and contribute to the maintenance of the nervous environment, involving microglia in dopaminergic neurodegeneration in the substantia nigra pars compacta and striatum by releasing proinflammatory factors [56]. Similarly, immune activation occurs in the whole process of HD, including microglia, monocytes, macrophages, and other primary abnormalities. The abnormal increase of IL-6 level in plasma in HD is the first discovery, but the nature of immune system activation is still unknown. It may be that the expression of mutant Huntington protein causes abnormal function of monocytes and lymphocytes that release IL-6 and activates NF-*κ*B signaling pathway to stimulate the release of IL-6, causing neurotoxicity [57, 58]. The relationship between the peripheral inflammatory response and the central nervous system may be the interaction between them through the blood-brain barrier by some regulatory factors. The study confirmed that the expression level of miR-9^∗^ in the brain and peripheral blood leukocytes of HD patients was significantly lower than that of healthy people, indicating that miR-9^∗^ may participate in the pathogenesis of HD by regulating the immune system of HD patients [59]. Other studies have found that miRNAs may regulate innate and adaptive immunity by affecting the production of type 1 and type 2 cytokines. At the same time, miRNAs such as hsa-miR-4488, hsa-miR-196a-5p, and hsa-miR-549a are highly expressed in HD, and miR-196a may regulate neuronal functional status through immune system, adaptive immune system, cytoskeleton remodeling, ABC transporters, etc. It is involved in the transcriptional regulation and translation inhibition of target genes in the development of HD disease, and the change of CAG trinucleotide repeat sequence length is related to the imbalance of miRNAs [60, 37, 61]. HD is caused by an increase in CAG trinucleotide duplication of the HTT, which leads to protein aggregation and leads to impaired motor and cognitive function in patients. Therefore, in the treatment of neurodegenerative disease such as HD, the noncoding characteristics of RNA and the interference technology of miRNAs are used to inhibit the expression of mutant HTT and other mutant proteins at the posttranscriptional level to improve the migration defect of immune cells [62–64].

Proven in the central nervous system that the abnormal accumulation of A*β* and *α*-syn activated Toll-like receptors (TLR) and NOD-like receptors (NLR) in microglia, Toll-like receptors are a class of protein molecules involved in innate immunity. TLR expression can be detected in astrocytes, microglia, and oligodendrocytes of both human and mouse [65, 66]. TLRs are highly abundant gene expression regulators in the central nervous system, belonging to the multigene family of immune system receptors. Some studies have shown that miRNAs released by apoptotic cortical neurons in neurodegenerative diseases, including miR-100-5p and miR-298-5p, interact with human TLR7 and/or TLR8 in microglia, resulting in neurodegeneration of mouse cerebral cortex and inflammatory response caused by aggregation of microglia [67]. Other studies have found abnormal expression of miRNAs and TLRs in AD, PD, ALS, and stroke patients. Previous studies have shown that let-7 of miRNA family is a signaling molecule of TLR7, which initiates the immune pathway of central nervous system (CD4+T cells) and participates in cell apoptosis. It is found that let-7 is significantly upregulated in cerebrospinal fluid of AD patients and is positively correlated with the expression of tau protein, which can be used as a biomarker and an auxiliary means to diagnose AD [68, 69]. In the brain of AD mouse model, miR-100-5p and miR-298-5p activate TLP7 in microglial cells, release proinflammatory factors, and damage neurons [67]. The activation of Nod-like receptor protein 3 inflammasome (NLRP3) has been detected in neurodegenerative diseases such as AD, PD, and ALS. Also in PD, *α*-syn activates NLRP3 inflammatory bodies in microglia cells, produces neuroinflammation, and aggravates PD. NLRP3 is a target gene of miR-7, which can directly inhibit the activation of NLRP3 inflammatory bodies induced by *α*-Syn and slow down the occurrence of neuroinflammation [66]. In neurodegenerative diseases, miRNAs can also aggravate or slow down disease progression by regulating microglial M1 and (or) M2. miR-125b activates NF-*κ*B signaling pathway by downregulating ubiquitin editing enzyme A20, so as to activate M1 microglial cells in ALS model, produce proinflammatory factors, and promote motor neuron apoptosis [70]. Research has shown that miR-155 is upregulated in the brains of AD patients and APP/PSEN1 mice after death, and a decrease in miR-155 may help alleviate pathology. miR-155 may mediate innate immune response components; A*β* amyloid pathway, synaptic pathology, and viral response gene network are involved in the therapeutic mechanism of neural degenerative disease [71]. It is found that miR-155 in microglia is abnormally increased in the SOD1 mouse model, ALS and AD brain. Through research, miR-155 activates M1-type microglia in SOD1 mice, leading to motor neuron damage, and by regulating the internalization of A*β* in microglia in AD, miRNA-155 can be used as a target gene for treating ALS and AD to improve inflammatory response [72, 73]. Similarly, miRNAs in the exosomes produced by inflammatory macrophages, such as miRNA-155-5p, reduce tyrosine hydroxylase (TH) positive cells in substantia nigra pars compacta and striatum and improve PD-like performance by inducing the activation of astrocyte and microglia. At the same time, miR-155 can also regulate the anti-inflammatory effect of miR-146a [73, 74]. miR-146a is considered to be the main regulator of innate immune response and inflammatory signals in human brain and central nervous system cells. Overexpression of miR-146a and miR-223 activates M2-type microglia, promotes phagocytosis of microglia, inhibits inflammatory response, and may improve accumulation of A*β*, clear myelin fragments, and promote myelin regeneration [75, 76]. At the same time, miR-146a can also regulate the inflammatory damage caused by astrocyte to neurons through the proinflammatory transcription factor NF-*κ*B, thereby improving the clinical symptoms of AD, ALS, and prion diseases, and may be used as an auxiliary treatment of neurodegenerative diseases and as a diagnostic biomarker [77–80]. It is known that miR-146a is an inhibitor of TLR. In myasthenia gravis (MG), miR-146a is downregulated and negatively correlated with levels of messenger RNA (mRNA) such as interleukin-1 receptor-related kinase 1 (IRAK1), tumor necrosis factor (TNF) receptor-related factor 6 (TRAF6), and inducible T cell costimulator factor (ICOS). This indicates that the downregulation of miR-146a may continue to activate TLR and inflammatory responses, as well as proliferative changes in the MG thymus, leading to the occurrence of MG. Therefore, we can speculate that increasing the level of miR-146a may regulate TLR, control autoimmunity, and improve the inflammatory performance of MG patients. In addition, miR-20b, miR-15a, miR-150-5p, miR-21-5p, and miR-30e-5p, as well as iR-151a-3p and miR-423-5p, are all abnormally expressed in MG and participate in the regulation of MG's autoimmune system [81–84].

In summary, the level of miRNAs in neurodegenerative diseases has changed and extracellular vesicles (EVs) can cross the blood-brain barrier and enter the blood. Therefore, miRNAs and some antibodies can be used as biomarkers of neurodegenerative diseases and can be used to detect whether the patient's nerves are degenerated through blood tests, which plays an important role in the diagnosis and treatment of diseases [85, 86].

### 2.2. miRNAs Regulate the Function of Stem Cells and Affect Neurodegenerative Diseases

At present, drugs for treating neurodegenerative diseases are emerging one after another, but there is still no effective treatment plan for this disease. In recent years, stem cells have been recognized as a new type of treatment for this type of disease, which can promote the regeneration of damaged nerve cells [87]. Stem cells have the ability of self-renewal, proliferation, and differentiation [88]. For example, by injecting umbilical cord blood stem cells into the aging rat model, it is found that the proliferation time of stem cells in the brain of aging rats is prolonged, the activation of microglia is reduced, and the microenvironment in the brain is improved [89]. Other experiments have shown that the injection of umbilical cord blood stem cells into PSAPP mouse model reduces CD40 ligand, thus reducing the accumulation of A*β* and improving inflammatory response [90]. At the same time, CD34 stem cells have also been used to treat PD model mice, improving motor activity, striatal dopamine, and ATP levels and playing a neuroprotective role [91]. However, the ability of stem cells to self-renew, proliferate, and differentiate is limited. When the body's neurons are damaged, beyond the stem cells' ability to self-repair, the body's nerve cells are damaged or even apoptotic, so the brain will have cognitive and motor dysfunction, memory and learning ability decline, and a series of neural degenerative disease [92].

It is reported that miRNAs can maintain the maturation of neural stem cells, promote the differentiation of stem cells into neurons, and repair the damage of brain nerves [93]. In order to prove that miRNAs regulate Dicer null NS cells to differentiate neural stem cells into neurons and/or glial cells, researchers used DICER ablation to eliminate the key proteins required by miRNAs and produced neural stem cell lines lacking all miRNAs. They found that these cell lines could not differentiate glial cells or neurons but had no effect on the self-renewal of neural stem cells; therefore, miRNAs are crucial for the survival and differentiation of stem cells in the nervous system [94]. In addition, miR-17-92 cluster maintains the appropriate number of cortical radial glial cells and intermediate progenitor cells by regulating PTEN and TBR2 proteins, thus promoting the proliferation and differentiation of neural stem cells and inhibiting neuronal apoptosis [95]. At present, neurotrophic factors, neuroinflammation, and miRNA signal transduction play an important role in the pathogenesis of ALS. Stem cells can produce growth factors and release anti-inflammatory cytokines to regulate inflammation and immune response and promote the repair and regeneration of damaged nerve cells. In the treatment of neurodegenerative diseases, the concentration of neurotrophic factor in cerebrospinal fluid increases significantly after autologous intrathecal injection of stem cells, and this neurotrophic factor has a protective effect on nerves [96]. Injecting autologous bone marrow-derived lineage-negative cells into the sheath of ALS patients, we found that neuroinflammation was alleviated and gene expression of neutrophils was reduced. At the same time, we found that the expressions of miR-16-5p, which has neuroprotective effects, and miR-206, which can promote muscle synaptic regeneration, were significantly increased, reducing motor neuron damage, which may play a role in improving the clinical symptoms of ALS patients [96–98]. Similarly, miRNAs can improve cognitive impairment caused by aging by promoting neurogenesis of neural stem cells. Overexpression of miR-153 can increase neurogenesis of neural stem cells, and its main mechanism is to regulate neurogenesis of hippocampal neural stem cells by inhibiting the translation of Jagged1 and Hey2, targeting Notch signal pathway [99].

Neural stem cells have strong neuronal regeneration and recovery abilities. The EVs they secrete reduce reactive oxygen species (ROS) and proinflammatory cytokines in brain cells, reducing the damage and death of dopamine neurons. At the same time, neural stem cell-specific miRNAs such as hsa-mir-9, hsa-let-7, and hsa-mir-183-5p have been found in EVs, which may be involved in the differentiation and immune regulation of neural cells in the brain of PD patients [100]. Another study has shown that mesenchymal stem cell-derived exosomes (MSC-EXOs) rich in miR-17-92 clusters may regulate the expression of PTEN protein and its downstream signaling pathway. PTEN protein controls cell apoptosis and affects kinases to promote protein phosphorylation, thereby preventing neuronal damage, increasing neural differentiation and plasticity after stroke, and promoting neural recovery [101]. Similarly, miR-223 has the function of inhibiting cell apoptosis. Studies have found a negative correlation between miR-223 and PTEN in AD, which may protect neurons by regulating the PTEN-PI3K/Akt pathway. Through research, miR-223 is downregulated in AD, and MSC-EXOs contain miR-223, which makes miR-223 upregulated in AD, reduces the apoptosis rate of neurons, and provides a potential therapeutic method for treating AD, PD, ALS, and other neurodegenerative diseases [102]. MSC-EXOs contain nucleic acids such as miRNAs, proteins, enzymes, lipids, and other compounds. It is a novel and rapidly developing cell-free therapy technology that can widely and effectively pass through the kynurenine to transfer biological materials between cells to alleviate and reduce neuronal damage and apoptosis. Some studies have found that mesenchymal stem cells (MSCs) are the only ones that can secrete MSC-EXOs in high yield. More interestingly, MSC has been able to effectively isolate from various sources such as bone marrow (BM), umbilical cord, Wharton's jelly, placenta, adipose tissue, or dental pulp [103, 104]. MSCs have a strong ability of immune regulation. They regulate the activation of microglia through cell contact and secreted EVs. Research has found that MSCs may reduce the activation of A*β* aggregates by blocking the upregulation of proinflammatory mediators such as TNF-*α* and nitric oxide (NO) [105]. Experiments showed that after injecting bone marrow mesenchymal stem cells (BM-MSCs) into the AD model with increased NF-*κ*B expression in the hippocampus, the inflammation of mice was reduced, and the level of miRNA-146a in the hippocampus was increased. In vitro experiments showed that miRNA-146a might promote the functional recovery of astrocytes, improve synaptic abnormalities, and correct cognitive disorders [31]. Neuroinflammation is also one of the factors leading to the degeneration of nerve cells in ALS. The gradual loss of motor neurons is the main pathological feature of ALS [106]. The experiment proved that miRNAs in EVs secreted by BM-MSCs can inhibit the pathological phenotype and neuroinflammation of astrocyte in ALS patients and SOD1G93A mice. In the experiment, SOD1G93A astrocytes were transfected with an EV shuttle miRNA synthesis mimic; researchers observed that seven out of nine miRNA mimics induced a significant reduction of GFAP, TNF-*α*, and IL-1*β* expression, and upregulated in IFN*γ*-primed MSCs, indicating that miRNAs have selective anti-inflammatory potential in SOD1G93A astrocytes. For example, after transfection with miR-29b-3p, the expression of NQO1 in astrocytes increased, and most MNs were repaired [107]. It has been reported that BM-MSCs have the ability to migrate to the injured site and promote the repair of injured neurons, but their own migration ability is limited. Studies have found that after successful transfection of miR-31 into BM-MSCs in vitro, the migration ability of BM-MSCs is enhanced, and its expression in cells is upregulated. miR-31 enhances the migration ability of BM-MSCs by increasing the expression of matrix metalloproteinase and CXC chemokine receptor type 4 protein [108]. In addition, extracellular vesicles secreted by adipose mesenchymal stem cells (ADMSCs) transfected with miRNA-22, miR-29c-3p, and miR-146a were transplanted into AD mice. It was found that the survival rate of neural cells increased, the expression of inflammatory factors decreased, ABACE1 expression was inhibited, and the Wnt/*β*-catenin pathway was activated, reducing the aggregation of A*β* amyloid protein and NF-*κ*B levels, effectively inhibiting cell apoptosis and inflammatory response in the brain of AD mice [108–111]. A large number of in vitro experiments have proved that miRNAs in the MSC-EXOs may play a huge potential for the treatment and improvement of neurodegenerative diseases. Through the above research, it is suggested that there may be some connection between miRNAs and stem cells, which plays a huge role in the occurrence and development of neurodegenerative diseases.

So far, exosomes have been proved to be a potential unit for the treatment of various difficult and miscellaneous diseases and will be the best alternative to cell therapy. In particular, MSC-EXOs have the original characteristics of mother cells; are widely distributed; exist in cells, tissues, and liquids; promote intercellular signal transmission; and have high regenerative potential and anti-inflammatory effect. The miRNAs contained in them can cross the kynurenine and enter the brain, compensating for the drawbacks of surgical procedures and medication, exerting advantages in various inflammatory and autoimmune diseases [112, 113]. More importantly, stem cells are more sensitive in pathological conditions and can migrate to pathological regions. miRNAs have strong specificity, and through their target genes, they regulate pathogenic factors and improve the pathological state of diseases. However, various experiments have confirmed that stem cells and miRNAs have certain positive effects on the repair of neurons in neurodegenerative diseases and the reduction of neuronal apoptosis. It is worth noting that although the application of stem cells in the treatment of various diseases has broad prospects, the purity of stem cell isolation, survival rate, migration rate after stem cell transplantation, heterogeneity of patients, and the presence of many elements in MSC-EXOs improved. Moreover, most of these studies are in vitro experiments and lack sufficient clinical trials to more effectively demonstrate the safety and effectiveness of stem cell therapy for neurodegenerative diseases. This will also pose a huge challenge for the future application of miRNAs and stem cells in the treatment of neurodegenerative diseases. In addition to releasing miRNAs to pathological regions, other compounds take advantage of this opportunity and have adverse effects on diseases or the therapeutic effects of miRNAs on diseases; these issues are currently under consideration. Most of these studies are in vitro experiments, and there is a lack of enough clinical experiments. The survival rate, migration rate, and whether other injuries are caused to patients after miRNAs transfected stem cells are transplanted into patients, which we do not know, will also be a huge challenge for the future use of miRNAs and stem cells to treat neurodegenerative diseases.

### 2.3. miRNAs Regulate Signal Pathways to Affect Neurodegenerative Diseases

Cell signal pathway refers to a series of enzymatic reactions that transmit extracellular molecular signals to cells and participate in various physiological and cell development processes. The abnormality of its transmission pathway can lead to various pathological reactions [114, 115]. Recent studies have found that the signal pathway plays an important role in the occurrence and development of neurodegenerative diseases. According to the measured miRNAs differentially expressed in the peripheral circulating exosomal of rats, 19 miRNAs target 766 mRNA, which are all related to aging. Recently, it was found that miRNAs regulate NF-*κ*B. The influence of signal pathways such as Nrf2, MAPK1, Notch, mTOR, AMPK, PTEN, and RelA/ApoE on neurodegeneration has been confirmed [116].

Previous studies have shown that the common characteristics of neurodegenerative diseases are neuroinflammation and the inhibition of energy metabolism and protein degradation function of neurons. The main reasons for these characteristics are the protein homeostasis disorder caused by oxidative stress, mitochondrial dysfunction, and neuroinflammation, which lead to the damage and death of neurons [117, 118]. let-7 is one of the first miRNAs to be found, and its expression changes with age. Once its expression is disordered, it will cause a variety of diseases, including neural degenerative disease. FoxOs and its downstream targets reduce protein toxicity and oxidative stress, and the accumulation of insoluble protein aggregates is crucial to protect neurons; it was found that hsa-miR-451a and hsa-let-7f-5p were downregulated in ALS, which may protect motor neuron cells from damage by regulating “FoxO signaling pathway,” “MAPK signaling pathway,” and “apoptosis” [119]. Transcriptional regulatory NF-*κ*B protein combines with the promoter of nuclear region to transcribe various proteins, which is a regulator of cell biological activity and plays an important role in autoimmune diseases, inflammation, and neurodegenerative diseases [120]. According to literature, the activation of the NF-*κ*B signaling pathway affects the aggregation of A*β* and tau pathology [121]. In degenerative disease, miR-146a regulates inflammation by targeting the key upstream signal protein of NF-*κ*B signaling pathway. In the experiment, the downstream expression of cytokines and chemokine messengers in mice lacking miR-146a significantly increased. Spleen enlargement and anxiety behavior are consistent with neuroinflammation in mice. Abnormal deposition of A*β* and neuronal fiber entanglement are two major pathological factors causing AD; A*β*1-42 is the main pathological component of A*β* in the early brain of AD. In this study, NF-*κ*B was infused into the hippocampus of rats and it was found that the NF-*κ*B pathway was activated; miRNA-146a-5p was upregulated, leading to a downregulation of IRAK-1 expression and a doubling of astrocytes. After treatment with the NF-*κ*B inhibitor BMS-345541, the expression of miRNA-146a-5p in astrocytes was significantly reversed, and IRAK-1 could be rescued, indicating that miRNA-146a-5p may pass NF-*κ*B pathway negative feedback loop which exerts anti-inflammatory function [122, 123]. Other studies have confirmed that miR-15b may directly target BACE1 and downregulate its expression by inhibiting NF-*κ*B activation, reducing the production and accumulation of A*β*, and reducing the secretion of inflammatory cytokines [124]. In addition, miR-22-3p can also act on Sox9 protein, which can regulate the differentiation of astrocyte and the expression of neural stem, through NF-*κ*B signaling pathway to reduce the abnormal aggregation of A*β* and improve the symptoms of AD [125]. HMGB1 (high mobility group box 1) is an important inflammatory factor, and research has found that HMGB1 is a target gene for miR-216a-5p. The expression of miR-216a-5p in the hippocampus of AD mice decreases, while the expression of HMGB1 protein increases. The NF-*κ*B p65 signaling pathway is activated in AD mouse cells. Research has confirmed that miR-216a-5p targets and inhibits HMGB1/NF-*κ*B pathway, reduces inflammatory response, slows down damage to hippocampal neurons, and improves learning and memory abilities in AD mice [126]. Similarly, in PD, microRNA-330 sponge may negatively regulate NF activity through target cell SHIP1, inhibit polarization of M1 microglia, repair dopaminergic neurons, and improve motor dysfunction in PD patients [127]. In addition, research has confirmed that downregulation of miR-218, miR-124, and miR-144 is associated with PD through activation of NF-*κ*B signaling [128]. Protein tyrosine phosphatase nonreceptor type 1 (PTPN1) is a target of A*β*-142-5p, involving in the regulation of synaptic and memory deficits in AD mice. Studies have shown that elevated levels of miR-142-5p, downregulation of PTPN1, and inactivation of the Akt pathway have been found in the brain tissue of AD rats. However, it was found in the study that lowering miR-142-5p and increasing PTPN1 improved the behavior of AD rats, indicating that downregulation of miR-142-5p targeting PTPN1 may activate the Akt pathway, thereby improving the learning and memory abilities of AD rats [129]. It has also been recorded that miR-124 is enriched in the brain and plays an important role in regulating the development, survival, and synaptic plasticity of neurons, previous studies have shown that miR-124 is overexpressed in the brain of AD model mice, and its translation is inhibited by directly targeting the 3′-UTR of PTPN1, altering the transmission of miR-124/PTPN1 signaling pathway signals, leading to the activation of GSK-3*β* and the inactivation of protein phosphatase 2A (PP2A), causing the occurrence of tau pathology and affecting synaptic transmission and plasticity, leading to memory impairment [130, 131]. Research has confirmed that miR-212-3p is significantly downregulated in AD, possibly regulating PDCD4 (a target protein of miR-212-3p involved in cell proliferation and apoptosis) through the PI3K/AKT signaling pathway and participating in the pathogenesis of AD [132]. Similarly, miR-539-5p, miR-107, and others were found to be significantly downregulated in AD patients and AD model mice but upregulated through their direct target GSK-3*β*. FGF2 regulates the PI3K/AKT/GSK-3*β* and FGFR2/PI3K/Akt signaling pathways, respectively, to protect neurons in AD from damage [133, 134]. However, miR-194 was significantly upregulated in the A*β*1-42-induced AD cell model, with its target gene Nrn1 downregulated. Nrn1 can promote regeneration and repair of the nervous system after damage, and overexpression activates the PI3K/AKT signaling pathway. Studies have shown that miR-194 may accelerate AD neuron apoptosis by inhibiting the activity of the PI3K/AKT pathway by inhibiting Nrn1 [135]. NLRP3, the promoter of the innate immune system, activates the NLRP3/Caspase-1 signaling pathway and prevents neuronal pyroptosis. It is known that overexpression of miR-212-3p in AD can reduce neuronal mortality. Previous studies have shown that miR-212-3p can target specific protein 1 (SP1), which enhances the expression of BACE1. In vitro experiments have shown that miR-212-3p can inhibit the activation of the NLRP3/Caspase-1 signaling pathway by targeting SP1 and BACE1, improving neuroinflammation in AD models [136]. SP1 is a multifunctional bioactive lipid, which is involved in regulating a variety of physiological and pathological mechanisms, including inflammation. AMPK is an AMP-dependent protein kinase, which plays a key role in bioenergy metabolism. Research has found that this signal pathway is involved in the inflammatory response of nerve degenerative disease and the survival of neurons in the hippocampus. Research has confirmed that miR-590-3 and SP1 play a crucial role in the regulation of AD genes, accelerating neuronal apoptosis by regulating the AMPK signaling pathway. SP1 is also associated with the AMPK signaling pathway as a target for miR-328-3p, a biomarker of AD [137, 138]. miR-200a is involved in regulating cell proliferation, differentiation, and cell cycle exit, which has a regulatory effect on striatum dopamine receptor D2 (DRD2), thus affecting the occurrence and development of PD. The main mechanism may be that miR-200a is upregulated in PD and promotes striatum cell apoptosis by downregulation of DRD2 and activation of cAMP/PKA signaling pathway [139]. There are reports that miRNAs regulate synaptic activity in AD model mice. miR-135a-5p is significantly downregulated in the hippocampus of AD mice and in the early stages of the frontal cortex of AD patients and is negatively correlated with the expression level of tau. It may be activated through the Rock2 signaling pathway, inducing synaptic disorders and leading to cognitive and memory impairment in mice. The activation of Rock2 leads to changes in dendrites and dendritic spines, and the targeted addition of protein 1 (Add1) exacerbates phosphorylation. Therefore, promoting the overexpression of miR-135a-5p is another new breakthrough in the treatment of AD [140]. PD is a type of dopaminergic neuron degeneration and protein *α*-synuclein (*α*-syn) clustering as a feature of motor disorders [141]. miR-342-3p has been found to be upregulated in PD, and PAK is its target gene. There are many literature records that PAK plays an important role in the cytoskeletal organization, cycle regulation, and survival of cells. The downregulation of miR-342-3p targets PAK1 to activate the Wnt signaling pathway, promote the expression of glutamate transporters and the proliferation of dopaminergic neurons, and improve the pathology of PD mice [142]. miR-124 is abnormally expressed in neurodegenerative diseases such as AD, ALS, and PD. Overexpression can improve the loss of dopaminergic neurons, improve motor deficits in PD mice, and is an important neuroprotective factor. Its mechanism is that miR-124 protects motor neurons and inhibits the progression of PD by inhibiting the negative regulatory factors Axin1 and Wnt/*β*-catenin signaling pathways of the Wnt pathway [143].

Under the environment of inflammation and mitochondrial dysfunction, it is easy to cause free radical aggregation to produce oxidative stress reaction. Oxidative stress reaction is a dynamic imbalance between the production and elimination of ROS. ROS accumulation promotes the formation of mitochondrial dysfunction, cell apoptosis, and protein misfolding in neurodegenerative diseases. The appearance of oxidative stress can regulate the production of free radicals to reduce neurotoxicity [144–146]. It is found that miRNAs are related to the regulation of intracellular redox state [118]. Nrf2 is the most important transcription factor for antioxidation and reduction in inflammatory environment. miRNAs can directly regulate the expression of Nrf2 protein, thereby affecting the homeostasis of ROS/RNS, strengthening the antioxidant capacity of nerve cells, reducing protein misfolding and accumulation, and improving the damage of neurons [147]. For example, miR-144 can regulate the expression of Nrf2, inhibit the production of antioxidant enzymes, and regulate the oxidative stress response, which may be part of the pathogenesis of AD. miR-25 may inhibit KLF2 and stimulate Nrf2 pathway. KLF2 is confirmed to be the target gene of miR-25, which has anti-inflammatory and antiatherosclerosis effects, thus aggravating A*β*1-42-induced hippocampal neuronal damage in AD mice [148, 149]. miR-592 is overexpressed in AD, which inhibits the antioxidant Keap1/Nrf2/ARE signaling pathway by downregulating its target gene KIAA0319, and promotes the oxidative stress injury of astrocytes in AD rat models. Mitogen-activated protein kinases (MAPKs) are serine threonine kinases that widely exist in the central nervous system and participate in intracellular signal pathways related to apoptosis, differentiation, and proliferation of various cells. In the AD model mice established by A*β*1-42 treatment, miR-191-5p was downregulated in their microglia and hippocampus. However, the overexpression of miR-191-5p reduced neuronal apoptosis. The mechanism may be that miR-191-5p inhibited the MAPK signal pathway of AD model microglia by downregulating Map3k12 and alleviated the damage of microglia [150]. In the PD mouse model, inhibiting the expression of miR-96 reduces the activation of iNOS by promoting CACNG5 inhibition of MAPK signaling pathway in PD mice, thereby reducing the damage of dopaminergic neurons and restoring the dysfunction of motor system [151]. More interestingly, miR-181a has been shown to be significantly downregulated in the PD model, and its overexpression can inhibit the activation of the p38MAPK/JNK pathway and reduce neuronal apoptosis [152]. In addition, research has confirmed that miR-326 can inhibit the activation and apoptosis of dopamine neuron iNOS by inhibiting the MAPK signaling pathway and negatively targeting KLK7. It can also target XBP1 to protect the function of dopaminergic neurons, inhibit the expression of iNOS by inhibiting JNK signals, promote the autophagy ability of PD mouse neuronal cells, and reduce damage to dopaminergic neurons [153, 154]. Another study suggests that activation of the MAPK signaling pathway can also lead to cognitive and memory disorders. Therefore, researchers speculate that the recovery of mitochondrial dysfunction in AD may be due to the overexpression of miR-330 inhibiting the MAPK signaling pathway, improving the incorrect accumulation of A*β*, and promoting the function of mitochondria to become normal [155]. Mitochondria are the main site of aerobic respiration, and their dysfunction plays a crucial role in the pathogenesis of AD. According to the literature, miR-98 can increase the level of mitochondrial DNA (mtDNA) in neurons of AD patients by inhibiting the activation of Notch signal and improve oxidative stress [156].

As the first line of immune defense in the brain, glial cells play an important role in clearing neurotoxic substances in AD. It is reported that miR-124-3p in microglial extracellular secretions from brain injured mice is promoted by targeting the RelA/ApoE signaling pathway *β*-amyloid hydrolysis and inhibitory A*β* aggregates, reduces neuroinflammation, improves hippocampal neuron damage, and alleviates neurodegeneration [157]. In HD, miR-124 can also protect the nerves from damage by regulating the activation of JAK/STAT3 pathway and activating ribonuclease 4 (RNASE4) to promote angiogenesis, neurogenesis, and neuronal survival, while promoting the reactivity of astrocytes and reducing the number of the Huntington condensates, improving and preventing the occurrence of HD [158–160]. Similarly, overexpression of miR-186 in AD inhibits the JAK-STAT signaling pathway and downregulates interleukin-2 (IL-2) to reduce inflammation, promote cell proliferation, and reduce A levels to improve hippocampal neuronal cell viability [161]. There is also a large amount of evidence that Akt/mTOR signaling pathway plays an important role in autophagy [162]. The deposition of A is the basis for the formation of amyloid plaques. Sirolimus target protein (mTOR) plays a key role in the regulation of autophagy. The deposition of A*β*1-40 is the basis for the formation of amyloid plaques. MTOR plays a key role in the regulation of autophagy. The activated mTOR inhibits the synthesis of autophagosome organisms, making the formation and elimination of the imbalance of amyloid plaques. In the AD model, there were significant differences in PI3K, p-AKT, and p-mTOR levels compared to the control group. At the same time, let-7b was upregulated, significantly reducing cell survival and inhibiting autophagy. The experiment confirmed that overexpressed let-7b induced PI3K/AKT/mTOR pathway to inhibit autophagy and promote cell apoptosis in AD model cells through A*β*1-40 [163]. Overexpression of miR-211-5p may target A*β* metabolism-related genes, reduce neuronal activity, and exacerbate the impact of neurotoxins on pathology. The target gene of miR-211-5p, Neurogenin 2 (Ngn2), is a transcription factor that promotes the differentiation of neural stem cells into neurons, and its levels are reduced in AD model cells. miR-211-5p may inhibit the activation of its downstream signaling pathway PI3K-Akt by targeting Ngn2, exacerbating neurodegeneration [128]. The overexpression of miR-33 promotes the secretion of antibodies through AKT/mTOR signal pathway and impairs the clearance of antibodies in nerve cells, leading to apoptosis of nerve cells and loss of synaptic plasticity-related proteins, thus aggravating the cognitive impairment of AD patients [164, 165]. 1-Methyl-4-phenyl-1,2,3,6-tetrahydropyridine hydrochloride (MPTP) is a fat soluble organic compound that can enter the brain through the blood-brain barrier and be metabolized into toxic 1-methyl-4-phenylpyridine (MPP+) by monoamine oxidase B in the brain. It has a toxic effect on the nerve cells that produce dopamine in the substantia nigra pars compacta of the brain, killing nerve cells and promoting PD pathological damage. As a promoter activated by ERK protein, miR-133b was significantly downregulated in PD cell models. When miR-133b was upregulated, the expression of ERK1/2 protein was significantly reduced. Research has shown that miR-133b may improve MPP+-induced apoptosis by inhibiting the activation of the ERK1/2 signaling pathway [166]. Similarly, research has confirmed that miR-593-3p accumulates in SH-SY5Y cells treated with MPP + and accelerates neuronal cell death. The mechanism is that miR-593-3p may weaken mitochondrial autophagy by inhibiting the PTEN-induced putative kinase 1 (PINK1)/Parkin pathway, accelerating neuronal death in PD [167]. PTEN plays an important role in regulating the autophagy ability of cells and is necessary for maintaining normal cell function in the nervous system. Studies have shown that both miR-4465 and miR-181b can inhibit the expression of PTEN to activate the AKT/mTOR signaling pathway and activate autophagy in cells [168, 169]. DEK is a highly conserved oncogene commonly present in value-added cells. Overexpression inhibits cell apoptosis, and DEK is a target gene for miR-138. Research has found that miR-138 is overexpressed in the AD model, with DEK significantly downregulated and AKT inactivated. The regulation of miR-138 may accelerate neuronal cell apoptosis by downregulating the DEK/AKT pathway [170]. The literature records that Kruppel-like factor 4 (KLF4) is not only the target gene of miR-212 but also the upstream regulator of Notch signaling pathway in a variety of biological processes. The literature records that Kruppel-like factor 4 (KLF4) is both the target gene of miR-212 and an upstream regulatory factor of Notch signaling pathway in various biological processes. Studies have confirmed that miR-212 inhibits Notch signaling pathway by targeting KLF4 in MPP+-induced SH-SY5Y cells, increases SOD and ROS levels, reduces the release of inflammatory factors such as TNF-*α* and IL-1*β*, increases motor neuron cell vitality, and alleviates MPP+-induced SH-SY5Y cell [171].

In addition, the study found that patients with diabetes (T2D) will increase the risk of suffering from neurological degenerative disease by more than twice, and neurological degenerative disease and diabetes have the common characteristics of insulin resistance (related to protein aggregation), activation of inflammatory response pathway, and abnormal autophagy function [172, 173]. Some studies have confirmed that miRNAs can also delay cellular senescence by regulating MTOR signaling pathway and insulin growth factor-1. MTOR and insulin/IGF-1 are important regulators of cell metabolism, proliferation, and survival. They can inhibit translation by regulating their mRNA, which may prevent the folding of wrong egg white and reduce the occurrence of neurodegenerative diseases. At present, the mechanism of miRNAs regulating MTOR and insulin/IGF-1 to inhibit aging is still uncertain [174–176].

Previous studies have shown that neuronal insulin signaling plays an important role in neurite growth, synaptic plasticity, mitochondrial function, and neuronal survival [177]. Other studies showed that anaplastic lymphoma kinase (ALK) and receptor-like tyrosine kinase (RYK) were downregulated by miR-1271, and Wnt/*β*-catenin signaling pathway upregulates the level of cell lineage specific TF paired box gene 4 (PAX4). PAX4 regulates the development and regeneration of pancreatic islets and is also related to neurodegenerative diseases. The upregulation of PAX4 promotes the expression of growth factor receptor bound protein 2 (Grb2) and NADPH oxidase 4 (NOX4) in AD and T2D. Similarly, in HD, insulin signaling can induce autophagy and reduce the abnormal aggregation of mutant huntingtin (mHtt). mHtt may block the neuronal insulin signaling pathway by inhibiting miR-302, resulting in insulin resistance and promoting HD and T2D pathology [172, 173].

However, studies have confirmed that the same miRNA has different effects on different diseases in the same organism and even leads to mutual inhibition between different diseases. For example, the increase of tumor suppressor miR-34c, which is differentially expressed in cancer and AD, can inhibit the occurrence of cancer and promote the progression of AD. The mechanism by which it promotes AD progression may be the upregulation of miR-34c, which exacerbates synaptic defects and affects cognitive function by mediating the ROS-JNK-p53 pathway targeting the prominent protein binding site 1 (SYT1) [178]. In addition, miR-196a has been found to be abnormally expressed in various malignant tumors and downregulated in AD. Upregulation of miR-196a may regulate the PI3/Akt pathway by downregulating its target gene LRIG3, inhibiting oxidative stress and inflammation damage, and inhibiting hippocampal neuronal apoptosis [179]. Therefore, there are significant challenges in the future development of regulating miRNAs for the treatment and prevention of diseases.

### 2.4. miRNA Regulates Intestinal Flora and Affects Neurodegenerative Diseases

The interaction of intestinal host microbiota maintains intestinal homeostasis. The intestinal microbiota is the largest microbiota of the human body and plays an important role in human metabolism, immune system, nutrient absorption, behavior, and cognition [180]. Recent research shows that TDP-43, a susceptibility gene of ALS, has obvious changes in the intestinal tract 10 years before the onset of symptoms in ALS patients, suggesting that gut microbiota has some relationship with the physiological and pathological mechanisms of neurodegenerative diseases [181]. It is understood that intestinal microbes interact with the central nervous system in a specific and complex way to form the so-called brain gut axis, which has an impact on the generation of newborn neurons in the hippocampus of adult mice and changes in their cognition and behavior, which is manifested in changes in neuronal morphology, neurogenesis, and serotonin neurotransmission. In an experiment between patients with cognitive impairment and healthy controls, significant differences were found in intestinal microbes between the two groups [182–184]. There are other reports that taking probiotics can improve the clinical symptoms of neurodegenerative diseases such as AD and PD. The treatment of various probiotics affects the composition of intestinal bacteria and the metabolism of serum tryptophan, causing a rebalancing of gut microbiota, which may help restore intestinal inflammation and activate the immune system and improve neurodegenerative diseases [185, 186].

c microbiota and brain. The literature shows that miRNAs from EVs can regulate the internal environment of the intestine. In the study, miR-181b-5p and miR-200b-3p can alleviate acute and chronic inflammation of the intestine and maintain intestinal stability by regulating M1 and M2 macrophages and gut microbiota, respectively [187]. In an experiment to study the effect of gut microbiota on miRNAs in the hippocampus of sterile (GF) mice, the specific pathogen free mouse gut microbiota was colonized in the intestines of GF mice, and the expression levels of miRNAs and mRNA in the hippocampus of GF mice were reduced [188]. It is suggested that miRNAs and gut microbiota often interact and regulate each other, and there may be a physiological and pathological mechanism by which the miRNA brain gut axis affects the body, opening up new avenues for new therapeutic targets. Previous studies have shown changes in the expression and sequence of miRNAs in irritable bowel syndrome, which is related to the brain gut axis. One study found that miR-510 and miR-342-3p were upregulated in IBS patients compared with healthy controls, and miR-29a was increased in their blood microbubbles, small intestine, and colon biopsies. In addition, miR-510 and miR-29a were found to target the 3′-UTR of the serotonin receptor type 3 subunit gene HTR3E and the 3′-UTR of the glutamine synthetase gene and regulate the expression of the serotonin receptor gene, glutamine synthetase, and intestinal membrane permeability, respectively. This indicates a certain relationship between miRNAs and the brain and intestines [8, 189]. It has been found that miRNAs exist in the intestines of humans and animals and regulate the microbiome in the intestines. It was found that miR-30d was enriched in the model of MS patients sclerosis and autoimmune encephalomyelitis. However, when their feces were transplanted into the recipient mice, it was found that the symptoms of the recipient mice were alleviated. At the same time, oral administration of synthetic miR-30d improved the symptoms of MS patients sclerosis by regulating the expression of lactase in Akkermansia muciniphila and increasing T cells [190]. According to research, miRNAs may affect the occurrence and development of neurodegenerative diseases by regulating gut microbiota and even play a key and decisive role. It is reported that miR-146b-5p has a high content in human brain neurons and immune cells. In AD patients and prion disease, miR-146b-5p was significantly upregulated in neocortex and hippocampus compared with the control group and interacted with the mRNA of AD, possibly by combining with the NF-*κ*B site, initiating the occurrence of inflammatory signals, causing inflammatory reaction, causing abnormal protein accumulation, and leading to progressive aggravation of neurodegenerative disease [191]. Studies have shown that dendritic cells (DCs) stimulated by gut microbiota secrete EVs, and overexpression of some miRNAs in EVs, such as miR-146b-5p and miR-155-5p, triggers immune responses by activating T cells to release proinflammatory factors [192]. After research, miR-155 is a noncoding miRNA that promotes the development of Th17 cells. Its expression is significantly reduced in gut-associated lymphoid tissue cells of AD model mice, suggesting that miR-155 may establish a close connection with the brain through intestinal immune effector cells [193]. There are literature records that lipopolysaccharides (LPS) from the gastrointestinal tract have been detected in the neocortex and neurons of the brains of elderly and AD patients. LPS is considered one of the proinflammatory neurotoxins, passing through the gastrointestinal tract into the systemic circulation and then crossing the blood-brain barrier into brain cells. Research has shown that LPS enters the systemic circulation and induces NF-*κ*B (p50/p65) activation through the blood-brain barrier, promoting proinflammatory miRNA-30b-5p, miRNA-34a, miR-146a, and miR-155. They bind to their target mRNA 3′-UTR, including mRNA encoding neuron specific neurofilament chain protein, which is similar to the structural changes of proteins in AD and is crucial for maintaining the cytoskeleton and shape, as well as for the transmission of synaptic information. Simultaneously, downregulated complement factor activates the complement system to release neurotoxicity and proinflammatory mechanisms [194–198]. The vesicles secreted by DCs can enter brain cells through the kynurenine due to their own properties. It can be seen that miRNAs, as a breakthrough point in the treatment of neurodegenerative diseases, may play a certain role in regulating the inflammatory response of the nervous system.

It is also documented that metabolic disorders have affected brain function. Tryptophan is an essential amino acid that regulates the stability of protein. Its metabolite kynurenine may have a certain impact on excitotoxicity neurotransmission, neuroinflammation, oxidative stress, amyloid aggregation, and neuronal function [199]. The study found that the brain gut axis system can affect the brain cell function by regulating tryptophan, serotonin, kynurenine, and short-chain fatty acid, the permeability of the blood-brain barrier, and the activity of peripheral immune cells and brain glial cells to improve the function of neural degenerative disease [200].

Canine uric acid pathway is the main pathway of tryptophan metabolism, which directly affects tau protein phosphorylation and A*β* deposition that cause AD [109]. Research shows that the key roles of miRNAs and intestinal microbiota in neural development overlap. The target gene of miRNAs is significantly excessive in the tryptophan-kynurenine pathway. The metabolism of kynurenine is related to a variety of neurological diseases. The concentration of kynurenine in sterile mice decreases, while the concentration of kynurenine tends to be normal after the normal intestinal flora settles. Therefore, miRNAs may regulate the metabolism of kynurenine by regulating the intestinal flora to improve the function of brain cells [201–203]. At present, the specific mechanism of miRNA regulation in the intestinal microbiota and brain remains to be fully studied. The research mechanism of intestinal flora affecting neurodegenerative diseases has been put on the agenda. It is believed that the application of miRNAs to regulate neurodegenerative diseases through intestinal flora as a target for treatment and prevention will provide more effective therapeutic approaches.

## 3. Clinical Trials

At present, people's understanding of degenerative disease and its impact on human beings urge researchers to urgently find new treatment methods and conduct intense research. The expression and dysfunction of miRNAs have been reported in various diseases, such as metabolic disorders, cancer, and nervous system disease. A large number of animal experimental studies have proved that miRNAs participate in cell growth, differentiation, and death, have diagnostic and therapeutic effects on neurodegenerative diseases, and make significant contributions to early clinical diagnosis and improvement of therapeutic effects. Nowadays, more and more clinical researchers have great interest in the study of the role of miRNAs in neurodegenerative diseases, designed a large number of clinical trials, and proved the positive role of miRNAs in clinical practice (Table 2). In clinical trials, researchers found that miR-107 and miR-206 may be regulated by A*β* precursor protein lyase 1 (BACE1) which reduces the abnormal accumulation of A*β* and improves the learning and memory ability of AD patients, and miRNAs can be used as a reliable biomarker for the diagnosis of AD (NCT01819545, NCT02129452) [204–206]. In the first human trial of AAV-mediated gene therapy in HD, AAV5 miHTT was found to degrade Huntington's protein and improve the microenvironment in HD brain (NCT04120493) [207]. These clinical trials have once again proved that miRNAs are involved in the occurrence and development of neurodegenerative diseases. Other clinical trials under study, such as drugs and other external stimuli, regulate the safety and effectiveness of miRNAs to prevent and improve AD (NCT02045056, NCT04840823), providing a broader exploration field for the future application of miRNAs in the treatment of neurodegenerative diseases. For early diagnosis of neurodegenerative diseases such as AD, except for abnormal protein accumulation imaging technology, few breakthroughs have been made. According to a large number of animal experiments, miRNAs can be used as biomarkers for early diagnosis of neurodegenerative diseases. A large number of clinical trials have been carried out to prove the differences between miRNAs in the brains of healthy elderly and AD patients, further proving that miRNAs can be used as biomarkers for early neurodegenerative diseases (NCT02253732, NCT02045056, NCT03388242, and NCT04137926).

However, some clinical trials have been conducted, but results have not yet been achieved. This may be due to the limited number of participants, the invasive nature of the testing methods, and the inability of most people to accept and compare the data (NCT01992029). There are still some clinical trials in preparation for recruitment and research to verify that miRNAs are biomarkers for diagnosing neurodegenerative diseases, but the results have not yet been released (NCT04961450, NCT03088839, NCT04137926, NCT02283073, NCT04509271, NCT02859428, NCT05243017). Although no results have been obtained, these clinical experiments provide important clues for finding new targets for the treatment of neurodegenerative diseases.

## 4. Summary

The emergence of miRNAs provides an effective way for the diagnosis and treatment of neurodegenerative diseases and plays an important role in brain diseases with its advantage of passing through the blood-brain barrier. At the same time, about one-third of human gene expression is regulated by miRNAs. A single miRNAs can interact with multiple genes, and the expression of miRNAs is highly specific [210]. miRNAs will be abnormal with the phosphorylation of tau protein, oxidative stress, neuronal damage, mitochondrial dysfunction, and other pathological damage. However, abnormal miRNAs will further aggravate the development of the disease. According to the changes of these miRNAs, they can be used as a target for disease diagnosis and treatment [136]. Therefore, miRNAs can not only treat diseases but also serve as biomarkers to diagnose diseases, making up for the shortcomings in the treatment and diagnosis of brain diseases. These advantages are being confirmed by a large number of animal experiments and clinical trials, providing broad prospects for the treatment of brain diseases. In neurodegenerative diseases, miRNAs regulate the immune system, stem cells, signal pathways, and intestinal flora to reduce the accumulation of incorrect proteins in the brain, improve the inflammatory response in the brain, and promote the repair and regeneration of damaged neurons. However, the mechanism of miRNAs regulating the microenvironment in the body is still unclear [211, 212]. Neurodegenerative disease is the focus of clinical and experimental research in recent years. Because it is a disease caused by multiple factors and the brain function is complex, the pathogenesis of most neurodegenerative diseases has not yet reached consensus, and the incidence rate is high, which seriously affects people's quality of life. Therefore, we need to further explore the pathogenesis of the disease and find a way to completely cure the disease.

The latest literature records that the novel coronavirus (SARS-CoV-2), which was once rampant, has a serious impact on degenerative disease, but its mechanism is still unclear. Research has found that in addition to common age issues, there are also common differentially expressed genes and miRNA expression abnormalities between the two, such as hsa-miR-155, hsa-miR-34a, and hsa-miR-132. In PD, hsa-mir-129-2-3p is a brain-enriched miRNAs that are elevated in the patient's peripheral lymphocytes and most significantly associated with the hub gene. It is suggested that miRNAs may become a potential therapeutic target for infectious diseases such as SARS CoV-2, swine flu, influenza, and the complications of nervous system disease caused by them [213, 214]. Although studies have found that specific miRNAs have excellent performance in the treatment and diagnosis of neurodegenerative disease, there are also some limitations. Most of the current studies are limited to in vitro experiments, and more are cell models or mouse pathological models. However, in the human brain, due to various factors such as genetics, environment, and emotion, the level of specific miRNAs is very low. If various methods are used to stimulate the level changes of specific miRNAs, miRNAs regulate multiple gene levels and may lead to harmful changes in necessary genes, leading to other pathological changes that may not occur in animal brains or cell models. Therefore, in the field of medical research, animal models cannot be compared to the human body itself [167]. In this review, although the role of miRNAs in degenerative disease has been elaborated in detail, the specific turnover mechanism and etiology are largely unclear, and its feasibility, effectiveness, and safety have not been ensured in clinical trials, which will become a major challenge in the field of scientific research.

## Figures and Tables

**Table 1:** Level changes, targets, signal pathways, and roles of miRNAs in degenerative disease.

Disease name	miRNA	State	Signaling pathway	Target spot	Research results
AD	miR-146a	Downregulation	NF-*κ*B↑	Unknown	Astrocyte↑ Inflammatory reaction↓

AD	miR-15b	Downregulation	NF-*κ*B↑	BACE1	A*β*↑ TNF-*α*↑ IL-1*β*↑ IL-6↑ COX-2↑ iNOS↑

AD	miR-22-3p	Downregulation	NF-*κ*B↑	SOx9	A*β*↑ Cognitive impairment↑

AD	miR-216a-5p	Downregulation	NF-*κ*B↑	HMGB1	TNF-*α*↑ IL-6↑ IL-1*β*↑

PD	miR-330sponge	Downregulation	NF-*κ*B↑	SHIP1	M1 microglia polarization↑ Microglia injury↑

AD	miR-142-5p	Upregulation	AKT↓	PTPN1	The learning and memory ability of AD↓

AD	miR-124	Upregulation	miR-124/PTPN1↓	PTPN1	Gsk-3*β*↑ PP2A↓ Tau pathology↑ Synaptic depletion and memory deficits↑

AD	miR-212	Downregulation	P13k/AKT↓	PDCD4	Cell proliferation↓ Apoptosis↑

AD	miR-539-5p	Downregulation	P13k/AKT↓	Gsk-3*β*	Damage to neurons↑

AD	miR-107	Downregulation	P13k/AKT↓	FGFR2	Damage to neurons↑

AD	miR-194	Upregulation	P13k/AKT↓	Nrn1	Damage to neurons↑

AD	miR-212-3p	Downregulation	NLRP3/Caspase-1↑	SP1, BACE1	IL-1*β*↑ IL-18↑

AD	miR-590-3	Upregulation	AMPK↑	SP1	Neuronal apoptosis↑

AD	miR-328-3p	Downregulation	AMPK↑	SP1	As a biomarker

PD	miR-200a	Upregulation	cAMP/PKA↑	DRD2	Striatal cell apoptosis↑

AD	miR-135a-5p	Downregulation	Rock2↑	Add1	Synaptic disorder↑ Tau pathology↑

PD	miR-342-3p	Upregulation	Wnt↓	PAK	Expression of glutamate transporter dopaminergic neurons↑

PD	miR-124	Downregulation	Wnt/*β*-catenin↓	Axin1	Injury of motor neurons↑

AD	miR-25	Upregulation	Nrf2↑	KLF2	IL-6↑ TNF-*α*↑ Hippocampal neuronal damage↑

AD	miR-592	Upregulation	Keap1/Nrf2/ARE↓	KIAA0319	Oxidative stress injury of astrocyte↑

AD	miR-191-5p	Downregulation	MAPK↑	Map3k12	Microglia injury↑

PD	miR-96	Upregulation	MAPK↑	CACNG5	iNos↑ Dopaminergic neuron damage↑

PD	miR-181a	Downregulation	p38MAPK/JNK↑	Unknown	Neuronal damage↑

PD	miR-326	Downregulation	MAPK↑	KLK7	iNos↑ Dopaminergic neuron damage↑
			JNK↑	XBP1	

AD	miR-330	Downregulation	MAPK↑	VAV1	A*β*↑ Mitochondrial disorder↑

AD	miR-98	Downregulation	Notch↑	HEY2	MTDNA↑ Improving oxidative stress

AD	miR-124-3p	Downregulation	RelA/ApoE↓	Rela	A*β*↑ Hippocampal neuronal damage↑

HD	miR-124	Downregulation	JAK/STST3↓	RNASE4	Angiogenesis↓ Neurogenesis↓ Neuronal damage↑ Reactivity of astrocyte↓ Aggregation of the Huntington protein↑

AD	miR-186	Downregulation	JAK/STST↓	IL2	A*β*↑ Damage to neurons↑

AD	let-7b	Upregulation	P13k/AKT/mTOR↑	Unknown	Cellular autophagy↓ Apoptosis↑

AD	miR-211-5p	Upregulation	P13k/AKT↓	Ngn2	A*β*↑

AD	miR-33	Upregulation	AKT/mTOR↑	Unknown	Antibody↑ Neurocyte apoptosis↑ Loss of synaptic plasticity-related proteins

PD	miR-133b	Downregulation	ERK1/2↑	ERK and AKT1	MPP+induced cell apoptosis↑

PD	miR-593-3p	Upregulation	PINK1/Parkin↓	PTEN	Mitochondrial autophagy function↓
PD	miR-4465	Downregulation	AKT/mTOR↑		Cellular autophagy ability↓
	miR-181b				

AD	miR-138	Upregulation	DEK/AKT↓	DEK	Neuron cell apoptosis

PD	miR-212	Downregulation	Notch↓	KLF4	TNF-*α*↑ IL-1*β*↑ SOD↓ ROS↓

PD	miR-185	Downregulation	P13k/AKT↓	IGF1	Autophagy of dopamine neurons↓ Neuron cell apoptosis↑

PD T2D	miR-1271	Downregulation	Wnt/*β*-catenin↓	ALK and RYK	PAX4↑ Grb2↑ NADPH↑

HD	miR-302	Downregulation	Insulin signaling pathway↓	Unknown	Insulin resistance mHtt↑ Mitophagy↓

AD	miR-34c	Upregulation	ROS-JNP-P53↑	SYT1	Outstanding defects

AD	miR-196a	Upregulation	P13k/AKT↑	LRIG3	Oxidative stress damage↑ Inflammatory reaction↑ Apoptosis of hippocampal neurons↑

↑: upregulation or activation; ↓: downregulation or transplantation; AD: Alzheimer's disease; PD: Parkinson; HD: Huntington's disease; T2D: type 2 diabetes; A*β*: amyloid-beta; PP2A: protein phosphatase 2A; MTDNA: mitochondrion; mHtt: mutant huntingtin; Add1: targeted addition of protein 1.

**Table 2:** Clinical trials of miRNAs in neurodegenerative diseases.

Study name (study date)	Current state	Study type	Subjects (age)	Purpose	Design	Interventions	*n*	Outcome measures	Research results
NCT02253732 (09/2015-03/2018)	Completed	Interventional	MCI AD PD HV (60-85)	Prevention	Open label	Exercise	30	miRNA myokines Cognitive function Motoric function	Exercise improved MDS-UPDRS, balance, walking speed, bradykinesia, REE, and glucose metabolism and increased the expression of AMPK and the expression of PRKAA1, MGF, Sirt1, Cox7a1, MyHC2, MyHC7, and other related genes increased [208].
NCT02045056 (05/2014-06/2019)	Completed	Interventional	AD (65-90)	Prevention	Double-blind placebo controlled parallel design	Gemfibrozil placebo	72	A E miRNA-107 A*β*1-40/1-42 FCSRT PAL	Not yet released
NCT03388242 (11/2017-03/2020)	Unknown	Observational	MCI (30-80)	Prevention	Open label	No intervention	120	(1) Fold changes of microRNAs in the blood of patients with MCI over control people. (2) Fold changes of proteins in the blood of patients with MCI over control people	Not yet released
NCT04137926 (03/2020-11/2022)	Recruiting	Interventional	AD HV (50-85)	Diagnostic	Double blind	miRNA battery	360	(1) miRNA battery for diagnostic of MCI due to AD. (2) miRNAs for intervene of MCI due to AD	Not yet released
NCT01819545 (01/2012-11/2015)	Unknown	Observational	AD (60-90)	Diagnostic	Open label	No intervention	300	Plasma and CSF press of miRNA 107 and BACE1 mRNA	(1) The overall classification accuracy of miRNA107 to discriminate between patients with amnestic mild cognitive impairment and healthy controls was 91.9%, with sensitivity of 98.3% and specificity of 82.7% [204]. (2) Alterations in key left brain regions associated with memory, language, and emotion in aMCI that were significantly correlated with plasma expression of miRNA107 and BACE1 mRNA [203]
NCT04120493 (10/2019-1/2021)	Recruiting	Interventional	HD (25-65)	Treatment	Double blind	Intrastriatal rAAV5-miHTT imitation (sham) surgery	26	AE AMT-130 fM UHDRS Q-Motor HD-CAB MRI HADS Neuro-QoL	Not yet released
NCT01992029 (06/2014-10/2015)	Terminated	Observational	Amyotrophic lateral sclerosis ALS (45-70)	Diagnostic	Open label	Clinical evaluation Muscular biopsy Lumbar puncture Blood sampling Neurological assessments Neuromuscular biopsy and lumbar puncture Blood sample Cervical spinal cord and brain MRI	5	miRNA expression	Not yet released
NCT03466723 (02/2019-06/2022)	Completed	Observational	PD (30+)	Diagnostic	Open label	Genetic: biomarker identification in Parkinson's disease	1000	Identification of novel circulating miRNAs as biomarkers for PD	Not yet released
NCT05055310 (10/2016-11/2019)	Completed	Observational	AD (60-90)	Diagnostic	Open label	Diagnostic test: miRNA test	1000	miRNA diagnosis validation for aMCI	Not yet released
NCT03217396 (11/2017-09/2027)	Recruiting	Observational	MS PD ALS AD (18-65)	Diagnostic	Open label	Procedure: lumbar puncture	300	Concentrations of CSF (neurofilaments, beta-amyloid, tau protein, inflammatory cytokines, and microRNAs)	In a large sample of MS patients that let-7b-5p levels in the cerebrospinal fluid (CSF) were highly correlated with a number of microRNAs implicated in MS, as well as with a variety of inflammation-related protein factors, showing specific expression patterns coherent with let-7b-5p-mediated regulation [209]

A*β*: amyloid-beta; AD: Alzheimer's disease; MCI: mild cognitive impairment; PD: Parkinson; HV: healthy volunteers; HD: Huntington's disease; AE: adverse events; FCSRT: free and cued selective reminding test; PAL: paired associate learning; BACE1: beta-site APP cleaving enzyme1; fM: CSF mutant protein; CSF: cerebrospinal fluid; UHDRS: Unified Huntington's Disease Rating Scale; Q-Motor: quantitative motor; HD-CAB: Huntington's Disease Cognitive Assessment Battery; MRI: magnetic resonance imaging; HADS: Hospital Anxiety and Depression Scale; MDS-UPDRS: clinical disability score; REE: resting energy expenditure; MGF: mechanogrowth factor.
